# Prospective evaluation of an automated method to identify patients with severe sepsis or septic shock in the emergency department

**DOI:** 10.1186/s12873-016-0095-0

**Published:** 2016-08-22

**Authors:** Samuel M. Brown, Jason Jones, Kathryn Gibb Kuttler, Roger K. Keddington, Todd L. Allen, Peter Haug

**Affiliations:** 1 Pulmonary and Critical Care Medicine, Intermountain Medical Center, Murray, USA; 2 Pulmonary and Critical Care Medicine, University of Utah School of Medicine, Salt Lake City, USA; 3 Clinical Intelligence and Decision Support, Kaiser Permanente Southern California, Pasadena, USA; 4 Homer Warner Center for Informatics Research, Intermountain Healthcare, Murray, USA; 5 Intensive Medicine Clinical Program, Intermountain Healthcare, Salt Lake City, USA; 6 Institute for Healthcare Delivery Research, Intermountain Healthcare, Salt Lake City, USA; 7 Shock Trauma ICU, Intermountain Medical Center, 5121 S. Cottonwood Street, Murray, UT 84107 USA

**Keywords:** Sepsis, Automated detection, Bayesian classifier

## Abstract

**Background:**

Sepsis is an often-fatal syndrome resulting from severe infection. Rapid identification and treatment are critical for septic patients. We therefore developed a probabilistic model to identify septic patients in the emergency department (ED). We aimed to produce a model that identifies 80 % of sepsis patients, with no more than 15 false positive alerts per day, within one hour of ED admission, using routine clinical data.

**Methods:**

We developed the model using retrospective data for 132,748 ED encounters (549 septic), with manual chart review to confirm cases of severe sepsis or septic shock from January 2006 through December 2008. A naïve Bayes model was used to select model features, starting with clinician-proposed candidate variables, which were then used to calculate the probability of sepsis. We evaluated the accuracy of the resulting model in 93,733 ED encounters from April 2009 through June 2010.

**Results:**

The final model included mean blood pressure, temperature, age, heart rate, and white blood cell count. The area under the receiver operating characteristic curve (AUC) for the continuous predictor model was 0.953. The binary alert achieved 76.4 % sensitivity with a false positive rate of 4.7 %.

**Conclusions:**

We developed and validated a probabilistic model to identify sepsis early in an ED encounter. Despite changes in process, organizational focus, and the H1N1 influenza pandemic, our model performed adequately in our validation cohort, suggesting that it will be generalizable.

## Background

Sepsis, especially in its more serious forms, severe sepsis and septic shock, is an important clinical and public health problem, accounting for approximately 750,000 hospital admissions in the USA annually and 20 % of intensive care unit (ICU) admissions. Furthermore, severe sepsis and septic shock are associated with high short-term mortality (18–40 %) [1, 2]. Rapid identification and initiation of treatment are considered critical for patient survival with sepsis, even if some controversy exists about the precise details of treatment protocols [3–5]. Early, appropriate treatment of sepsis is widely recommended by the academic societies and has long been advocated by the inter-society Surviving Sepsis Campaign [6].

### Importance

Given the limited time during which to affect the course of sepsis, early identification of severe sepsis or septic shock is crucial. Early, automated identification of severe sepsis or septic shock could potentially a) decrease the time to advanced diagnostics and risk assessment, b) decrease time to receipt of appropriate treatment, such as antibiotics, volume expansion, and vasopressors, c) enhance quality improvement efforts, and d) assist with timely study enrollment for septic patients.

### Goals of this investigation

In order to facilitate early identification, we sought to develop a probabilistic model to identify septic patients within one hour of entering the emergency department (ED) as part of a broader effort to improve compliance with Surviving Sepsis Campaign sepsis management bundles at Intermountain hospitals [7]. We aimed to develop a system that demonstrated good sensitivity with a clinically tolerable false positive rate.

## Methods

### Study design and setting

As we reported in abstract form, we developed the model using a data set that included all Emergency Department (ED) encounters (132,748 encounters, of which 549 were septic) from January 2006 through December 2008 from both LDS Hospital and Intermountain Medical Center [8]. In that retrospective study, data available within 1 h of ED arrival (temperature, mean blood pressure, heart rate, and white blood cell count) were used for a model with an area under the curve (AUC) of 0.836. The prospective validation we report here covered April 2009 through June 2010 and was conducted at Intermountain Medical Center. Intermountain Medical Center is a 456-bed teaching hospital affiliated with the University of Utah School of Medicine which replaced LDS Hospital in October 2007 as Intermountain Healthcare’s tertiary referral center and Level One trauma facility. (LDS Hospital remained in operation, transitioning into a secondary referral center status.)

### Selection of participants

Patients were identified as having severe sepsis or septic shock based on manual chart review by trained data and chart abstractors of patients with compatible International Classification of Disease-9 (ICD-9) discharge diagnosis codes as part of a broader quality improvement effort [9]. Patients were selected as candidates for chart abstraction if they were admitted directly from the ED to the intensive care unit (ICU) and either had a discharge diagnosis code related to sepsis or infection or were identified separately by quality improvement coordinators in the ICU. The ICD-9 codes were chosen to include broad representation of diagnoses associated with sepsis or infection and thus to maximize sensitivity with the knowledge that chart review would subsequently minimize potential false positive results. Encounters for all patients 14 years of age or older at the time of the encounter were included in the prospective validation.

### Methods and measurements

A chart review datamart was populated daily based on discharge diagnoses with subsequent chart review by trained data collectors. In addition to determining whether patients met consensus criteria [10] for severe sepsis or septic shock, data collectors also determined whether care was compliant with the Intermountain sepsis management bundles. Results of the compliance with sepsis bundles among septic patients have been reported separately [9].

To support improved compliance and quality of care as part of the overall bundle-driven quality improvement effort, we developed the probabilistic model whose accuracy is described in this report in both the retrospective (reported previously) and prospective models. Clinician collaborators initially identified 75 parameters that they suspected might be useful in the rapid identification of septic patients. These included basic demographic information, encounter details (e.g., time of day, day of week, method of arrival in the ED), results of laboratory tests, vital signs, other clinical measures, and the coded chief complaint. For any parameter for which multiple measures were available, the first available measurement in the ED was preferred. The components of the standard definition of the Systemic Inflammatory Response Syndrome (SIRS) [10] (e.g., heart rate greater than 90 beats per minute) were also included as candidates within the model. Clinicians also described desired performance characteristics for the model based on their *a priori* beliefs about acceptable sensitivity and false positive rates.

### Alert deployment

Chart review from January 2006 through December 2008 was completed in January 2009. During February and March 2009, the model was reviewed for overall performance characteristics (e.g., sensitivity, false positive rate) and individual case review by clinicians to determine whether performance was satisfactory and at what threshold to trigger an alert. The decision was made to implement the sepsis alerting system and a binary threshold was selected (0.05, though known calibration issues with naïve Bayes models do not support a simple interpretation of that threshold).

The alert system consisted of (a) routine clinical data entry, (b) pushing the data to a research decision support environment, (c) generating a probability estimate using Netica™ (Norsys Software Corp), a tool for developing/deploying Bayesian systems using data available at 60 min from ED entry, and (d) sending a page and email to the ED charge nurse for clinical consideration and action if the threshold was met. While training of charge nurses and other clinicians was ongoing throughout the process, we did not explicitly measure the effect of training on model performance.

### Changes between model development and validation

There were several differences between the time periods of model development and model validation. Organizational changes included the movement of the primary center from LDS Hospital to Intermountain Medical Center midway through the development period and the implementation of the alerting system after the development period. From a system perspective, both the triage nurse identification system (described below) and the model-based alert were deployed after the development period. After initial implementation, the model-based alert was shifted from 60 min post ED admission to 90 min post ED admission in order to capture more laboratory results. Finally, the H1N1 influenza pandemic [11] occurred during the model validation period and after model development.

Throughout these changes, the same data processes and procedures were maintained for the source data, chart abstraction, chart review, and model assessment.

### Triage nurse identification system

To maximize the chances of rapid patient identification, a parallel effort was made to identify sepsis patients via a modification to the triage nurse screens in the hospital electronic medical record. This system prompted the nurse to consider whether the patient had a new infection if the patient displayed fever, tachycardia, hypotension, or tachypnea. The triage nurse information was not considered as a potential predictor for the model, as it was not available during the model development phase. Data on the triage nurse information are provided for comparison purposes only.

### Outcomes

The outcome of interest for model development was confirmed diagnosis of severe sepsis or septic shock. For assessment of model performance, the outcome was sensitivity > 80 % and a false positive rate < 15 alerts per day.

### Analysis

The methodological details of the statistical modeling environment will be described in a separate publication. Briefly, it is a naïve Bayes framework that employs forward stepwise selection based on out-of-bag area under the receiver operating characteristic curve (AUC) derived via bootstrap sampling [12, 13]. The naïve Bayes model was selected due to substantial, non-random missingness (especially in laboratory values) and a desire to avoid imputation methods. Naïve Bayes deals with non-random missingness natively and performs better than traditional regression methods at non-random missingness. Briefly, a missing variable does not affect the overall probability estimate of the model. Additionally, the modeling environment allows for direct clinician input with respect to preferences for certain parameters over others—should certain parameters be of statistically equivalent utility but more easily attained, for instance. Among highly collinear variables the naïve Bayes model we employed selected the variable most predictive of outcome. Because we were interested in prediction rather than model inference, we therefore performed no analyses of collinearity. In a naïve Bayesian model, parameters do not have weights/coefficients; instead each parameter is associated with a mean and standard deviation in septic and non-septic patients, and the differences in these distributions are used to build the prediction model.

All model development was based on the R statistical computing environment [14].

### Model performance assessment

The primary success measures specified by clinicians were sensitivity (target 80 %) and the false positive count (target <15 patients per day). Based on ED visit volume, the target false positive rate equated to approximately 7.2 %. We calculated the AUC for both the continuous prediction model and the binary alert. For the binary alert, the positive predictive value (PPV), negative predictive value (NPV), and incremental (compared to the triage nurse system) true positive counts were calculated.

Final probability estimates from the model were calculated in Netica and then incorporated into the alerting environment; all other analyses were conducted with R.

## Results

### Characteristics of study subjects

From April 2009 through June 2010 (15 months) there were 93,773 ED encounters involving 58,603 unique patients at Intermountain Medical Center. Table 1 presents patient characteristics with the encounter as the unit of analysis for those encounters not involving sepsis (*n* = 93,421; 58,269 unique patients) and those involving sepsis (*n* = 352 [0.38 %]; 334 unique patients). The only parameter not significantly different across groups was gender (58.2 % female for non-septic encounters and 53.7 % for septic encounters). For encounters involving sepsis, patients tended to be older (median 60.9 versus 39.6 years), had a lower blood pressure (median 109/60 versus 132/77 mmHg) within one hour of entering the ED, a higher temperature (median 37.7 versus 36.4 Celsius), a higher heart rate (median 111 versus 86 min^−1^), and higher white blood count (14,300 versus 8,500 μL^**−1**^). Measurement rates were similar for vital signs (> = 95 %) but WBC was drawn more frequently within one hour of ED admission for encounters involving sepsis (53 % versus 22 %).

**Table 1:** Basic encounter characteristics at 1 h from ED entry

	Non-Septic Encounters				Septic Encounters				*p*-value
	Pt. Est.	20 %	75 %	% Avail	Pt. Est.	20 %	75 %	% Avail	
n	93,421	(58,269 unique patients)			352	(334 unique patients)			
Age	39.6	27.3	57.1	100 %	60.9	51.7	73.4	100 %	0.0000
Female	58.2 %	57.9 %	58.5 %		53.7 %	48.3 %	59.0 %		0.0934
SBP	132	119	146	97 %	109	91	129	97 %	0.0000
DBP	77	68	86	97 %	60	48	73	97 %	0.0000
Mean BP	95	86	105	97 %	75	64	94	97 %	0.0000
Temp (C)	36.4	36.0	36.8	95 %	37.7	36.6	38.7	96 %	0.0000
HR	86	75	99	97 %	111	91	126	98 %	0.0000
WBC	8.5 K	6.7 K	10.9 K	22 %	14.3 K	9.8 K	20.6 K	53 %	0.0000
Lactate	1.7	1.2	2.5	3 %	2.8	1.9	4.7	36 %	0.0000
ᅟ									
Screen	6.8 %	6.6 %	6.9 %		61.9 %	56.6 %	67.0 %	0	0.0000
Flag	3.1 %	3.0 %	3.2 %		54.3 %	48.9 %	59.6 %	0	0.0000

Median and quartiles provided for continues variables

Mean and 95 % exact confidence intervals provided for binary variables

*SBP* Systolic blood pressure, *DBP* Diastolic blood pressure, *BP* blood pressure, *HR* heart rate, *WBC* white blood cell count

### Main results

#### Model components

In the retrospective work reported previously and the prospective validation reported here, we included five parameters: temperature, heart rate, respiratory rate, white blood cell count, and mean arterial pressure.

#### Overall model performance

**Fig. 1:**
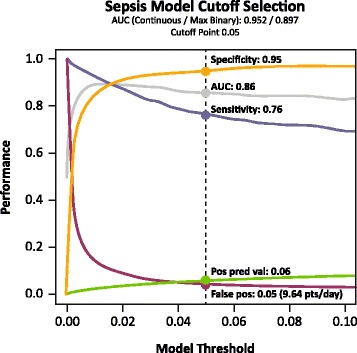
Model threshold selection based on overall performance characteristics

Table 2 and Fig. 1 present the prospective performance characteristics of the model, various alerting thresholds, and the two alternate alerting approaches (triage nurse and SIRS-derived rule) over the 15-month period. The AUC for the continuous predictor was 0.953. The pre-selected threshold for alerting was at a model generated probability of 0.05. This generated an AUC of 0.859, a sensitivity of 76.4 %, and a false positive rate of 4.7 % (median 9.64 false positive alerts per day). The triage nurse identification process generated a lower AUC (0.756), sensitivity (54.3 %), and false positive rate (3.1 %). The SIRS-derived rule generated even lower measures (AUC = 0.606, sensitivity = 21.6 %, false positive rate = 0.4 %).

**Table 2:** Overall performance characteristics

Alert	Cutoff	sens	fpr	auc	spec	ppv	npv	FP/Day	Inc. Sepsis TP	
									Nurse	Other
Triangle Nurse	1.000	54.3 %	3.1 %	0.756	96.9 %	6.1 %	99.8 %	6.44		
SIRS Derived	1.000	21.6 %	0.4 %	0.606	99.6 %	17.2 %	99.7 %	0.80	187	4
**Cut05**	**0.050**	**76.4 %**	**4.7 %**	**0.859**	**95.3 %**	**5.8 %**	**99.9 %**	**9.64**	**22**	**100**
Cut04	0.040	79.0 %	5.4 %	0.868	94.6 %	5.2 %	99.9 %	11.16	18	105
Sens80	0.036	80.1 %	5.8 %	0.871	94.2 %	4.9 %	99.9 %	12.03	17	108
FPC15	0.026	85.2 %	7.3 %	0.890	92.7 %	4.2 %	99.9 %	15.00	13	122
MaxAUC	0.017	89.2 %	9.9 %	0.897	90.1 %	3.3 %	100.0 %	20.33 %	12	135
NrsFP	0.097	70.2 %	3.1 %	0.835	96.9 %	7.8 %	99.9 %	6.44	32	88
NrsSens	0.330	54.3 %	1.6 %	0.763	98.4 %	11.4 %	99.8 %	3.27	64	64

*SIRS* systemic inflammatory response syndrome, *Cut05/Cut04* Bayesian cutpoint threshold of 0.05/0.04, *Sens80* threshold for sensitivity = 80 %, *FPC15* threshold of 15 false positive alerts per day, *MaxAUC* threshold maximizing area under the receiver operating characteristic curve, *NrsFP* nurse-driven false positive threshold, *NrsSens* nurse-driven sensitivity threshold. Boldface indicates the primary cutpoint used

The pre-specified goals for the alerting model were 80 % sensitivity and fewer than 15 false positives per day (false positive rate ~7.2 %). We identified different thresholds to achieve both of these targets. Selecting an alerting threshold of 0.036 would have achieved a sensitivity of 79.0 % (at goal) and false positive rate of 5.4 % (better than goal). Selecting an alerting threshold of 0.026 would have achieved a sensitivity of 85.2 % (better than goal) and false positive rate of 7.3 % (at goal). AUCs for the respective model are displayed in the Table.

Because the triage nurse identification process ran concurrently with the probabilistic sepsis alert model, it is possible to evaluate whether the two systems identified the same patients. The last two columns in Table 2 indicate how many of the 352 sepsis encounters were uniquely identified only by either the triage nurse process or the sepsis alerting model. Using the pre-specified cutoff of 0.05, the triage nurse contributed an incremental 22 correct alerts, while the model contributed 100. Had the model threshold been set at 0.026 (allowing a false positive rate of 7.2 %), the triage nurse would have added 13 alerts and the model would have added 122.

#### Performance by month

**Fig. 2:**
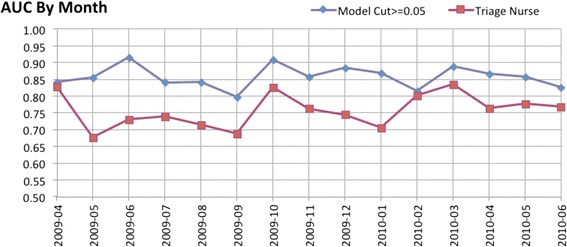
Monthly area under the curve for sepsis alert model and triage nurse

The number of ED encounters, rate of sepsis, and performance characteristics were evaluated by month (Table 3, Fig. 2). Overall, 0.38 % of encounters involved sepsis, with a range of 0.27 to 0.47 % and a coefficient of variation (CV) of 16.72 %. The continuous predictor had an overall AUC of 0.953; with a range of 0.931 to 0.974, and a CV of 0.014 (the lowest of any measure). The sensitivity of the 0.05 threshold alert model ranged from 64 to 89 %, while its false positive rate ranged from 4.2 to 5.6 %. The triage nurse system demonstrated higher variability by month. Sensitivity ranged from 38.1 to 70.6 % and the false positive rate ranged from 2.3 to 4.1 %. In general the CV for the triage nurse process was roughly twice that of the model-based alert.

**Table 3:** Monthly performance characteristics

Total:	93,773	208	352	0.38 %	Cont. AUC	Model Cut > =0.05				Triage Nurse			
Month	Encs	Per Day	Sepsis	Rate		Sens	FPR	FP/Day	AUC	Sens	FPR	FP/Day	AUC
200904	6,279	209	22	0.35 %	0.957	72.7 %	4.5 %	9.43	0.841	68.2 %	2.7 %	5.69	0.827
200905	6,583	212	21	0.32 %	0.952	76.2 %	5.2 %	11.00	0.855	38.1 %	2.8 %	5.86	0.677
200906	6,860	229	26	0.38 %	0.963	88.5 %	5.6 %	12.82	0.914	50.0 %	4.1 %	9.27	0.730
200907	6,736	217	22	0.33 %	0.974	72.7 %	4.6 %	10.03	0.841	50.0 %	2.3 %	4.92	0.739
200908	6,439	208	22	0.34 %	0.934	72.7 %	4.3 %	8.97	0.842	45.5 %	2.5 %	5.18	0.715
200909	6,352	212	25	0.39 %	0.961	64.0 %	4.6 %	9.64	0.797	40.0 %	2.6 %	5.59	0.687
200910	6,451	208	29	0.45 %	0.957	86.2 %	4.5 %	9.43	0.908	69.0 %	3.6 %	7.49	0.627
200911	5,975	199	25	0.42 %	0.953	76.0 %	4.5 %	8.94	0.858	56.0 %	3.5 %	7.00	0.762
200912	5,753	186	27	0.47 %	0.944	81.5 %	4.7 %	8.75	0.884	51.9 %	3.0 %	5.51	0.744
201001	6,206	200	27	0.44 %	0.932	77.8 %	4.2 %	8.49	0.868	44.4 %	3.4 %	6.71	0.705
201002	5,568	199	22	0.40 %	0.948	68.2 %	5.1 %	10.11	0.815	63.6 %	3.5 %	6.96	0.801
201003	6,250	202	17	0.27 %	0.965	82.4 %	4.6 %	9.28	0.889	70.6 %	3.8 %	7.63	0.834
201004	5,885	196	27	0.46 %	0.947	77.8 %	4.4 %	8.71	0.867	55.6 %	2.9 %	5.73	0.763
201005	6,184	199	17	0.27 %	0.969	76.5 %	4.9 %	9.80	0.858	58.8 %	3.4 %	6.79	0.777
201006	6,252	208	23	0.37 %	0.931	69.6 %	4.4 %	9.10	0.826	56.5 %	3.0 %	6.19	0.768
			Min:	0.27 %	0.931	64.0 %	4.2 %	8.49	0.797	38.1 %	2.3 %	4.92	0.677
			Max:	0.47 %	0.974	88.5 %	5.6 %	12.82	0.914	70.6 %	4.1 %	9.27	0.834
			CV:	16.72 %	0.014	8.7 %	7.9 %	0.11	0.038	18.8 %	16.4 %	0.18	0.066

#### Practical observations during model development

During the development phase we found that clinicians tended to reject alerts based solely on vital signs (i.e., lacking laboratory indicators). Many clinicians expected serum lactate to be a useful distinguisher, but this was not the case because serum lactate was available for <5 % of non-septic patients within a one-hour timeframe. White blood cell count (WBC) was found to be useful in the model development phase, though it was only available approximately 20 % of the time. Due to the reluctance of clinicians to accept alerts without elevated WBC, the decision was made to delay the alert from 1 to 1.5 h post ED entry. Technical performance did not improve appreciably with the modification, but communication with clinicians (and trust in the alert) improved substantially.

The primary limitation of this study is the fact that validation was conducted at a single center. Mitigating this limitation is the fact that many of the cases used to develop the model came from a different hospital from the one used for the prospective validation. Additionally, there were enough contextual differences between model development and validation that we are less concerned about the generalizability of the results. There are some limitations that may constrain the export of this sepsis alert model to other institutions. First, our system requires electronic charting of ED triage, vital signs, and laboratory test results. Second, measurement of the performance characteristics of this model requires chart review to confirm the presence of sepsis. This limitation could be obviated by only requiring periodic tests of calibration. We also acknowledge that our case-finding strategy was limited to cases of severe sepsis and septic shock that required ICU admission. While we felt it important to focus on those higher-risk patients, the model will require independent validation among patients with severe sepsis who do not require ICU admission.

## Discussion

Though early identification has been suggested as a key step in treating sepsis and decreasing sepsis-associated mortality, there is little information on how to efficiently and accurately identify septic patients in a usual care setting consistent with typical workflows. The results reported here demonstrate that it was possible to build a probabilistic model to identify septic patients and select an alerting threshold to achieve the pre-specified performance goals of 80 % sensitivity and a ~7.2 % false positive rate. The selected alerting threshold for the model was close to but missed the target sensitivity while performing better than the false positive target.

We also intended this study to demonstrate the process by which such an alert could be deployed. One of the biggest issues facing any alert system is “alert fatigue” [15]. The decision to deploy this particular sepsis alert model came in the context of a failed prior attempt in which performance of a rule-based alerting tool was over-estimated and an initial roll-out of the triage nurse process that generated too many false positives and lead to the disengagement of clinicians. Our decision to use the 0.05 cutoff, intended to avoid alert fatigue, meant that observed sensitivity was just short (76 %) of the pre-specified sensitivity threshold (80 %).

While aided by organizational factors such as the concurrent quality improvement effort, we believe that the implementation of this sepsis alerting system was facilitated by clear performance target setting and individual case review. In particular, the targets were set in terms meaningful to clinicians: 8 out of 10 sepsis patients identified and <15 false alerts per day. The latter is particularly dependent upon local circumstances (e.g., propensity to alert fatigue, ED load, staffing, institutional culture) in addition to the obvious dependence on institutional volumes. While the AUC commonly used to assess the discrimination of predictive models suggested that our model performed well, we believe that the sensitivity and false positive rate are more relevant indicators for ED clinicians.

## Conclusions

We have demonstrated that a probabilistic sepsis alert model can be deployed in a large hospital ED to identify sepsis patients according to the goals of 80 % sensitivity and a false positive rate of ~7.2 %. Additionally, it appears that the developed model behaved as expected despite significant contextual changes between the model development and validation periods. Though measures such as the positive predictive value and incremental patients identified by the described triage nurse process recapitulate the need for engaged clinicians in any form, we believe this alert approach can offer an opportunity for solid, stable augmentation of diagnostic processes relevant to the early detection of sepsis in the ED. Finally, the ability to tune alerting thresholds may facilitate balancing local goals and tolerances for error.

## Abbreviations

AUC: Area under the receiver operating characteristic curve
CV: Coefficient of variability
ED: Emergency department
ICD-9: International classification of diseases-9
ICU: Intensive care unit
NPV: Negative predictive value
PPV: Positive predictive value
SIRS: Systemic inflammatory response syndrome
